# Dynamic changes in genetic diversity, drug resistance mutations, and treatment outcomes of falciparum malaria from the low-transmission to the pre-elimination phase on the islands of São Tomé and Príncipe

**DOI:** 10.1186/s12936-021-04007-3

**Published:** 2021-12-14

**Authors:** Ying-An Chen, Tsen-Ju Shiu, Lien-Fen Tseng, Chien-Fu Cheng, Wei-Liang Shih, Arlindo Vicente de Assunção Carvalho, Kun-Hsien Tsai

**Affiliations:** 1 Institute of Environmental and Occupational Health Sciences, College of Public Health, National Taiwan University, Taipei, Taiwan; 2 Institute of Epidemiology and Preventive Medicine, College of Public Health, National Taiwan University, Taipei, Taiwan; 3 Taiwan Anti-Malaria Advisory Mission, São Tomé, São Tomé and Príncipe; 4 Infectious Diseases Research and Education Center, Ministry of Health and Welfare and National Taiwan University, Taipei, Taiwan; 5 Centro Nacional de Endemias, Ministério da Saúde de São Tomé e Príncipe, São Tomé, São Tomé and Príncipe; 6 Department of Public Health, College of Public Health, National Taiwan University, Taipei, Taiwan

**Keywords:** São Tomé and Príncipe, Malaria, *Plasmodium falciparum*, Merozoite surface proteins, Antimalarial drug resistance mutations, Recurrence

## Abstract

**Background:**

With effective vector control and case management, substantial progress has been made towards eliminating malaria on the islands of São Tomé and Príncipe (STP). This study assessed the dynamic changes in the genetic diversity of *Plasmodium falciparum*, the anti-malarial drug resistance mutations, and malaria treatment outcomes between 2010 and 2016 to provide insights for the prevention of malaria rebounding.

**Methods:**

Polymorphic regions of merozoite surface proteins 1 and 2 (*msp1* and *msp2*) were sequenced in 118 dried blood spots (DBSs) collected from malaria patients who had visited the Central Hospital in 2010–2016. Mutations in the multi-drug resistance I (*pfmdr1*), chloroquine resistance transporter (*pfcrt*), and kelch 13 (*pfk13*) genes were analysed by polymerase chain reaction-restriction fragment length polymorphism (PCR–RFLP) and sequencing in 111 DBSs. A total of 7482 cases that completed a 28-day follow-up were evaluated for treatment outcomes based on the microscopic results. Regression models were used to characterize factors associated with levels of parasite density and treatment failures.

**Results:**

Parasite strains in STP showed significant changes during and after the peak incidence in 2012. The prevalent allelic type in *msp1* changed from K1 to MAD20, and that in *msp2* changed from 3D7/IC to FC27. The dominant alleles of drug-resistance markers were *pfmdr1* 86Y, 184F, D1246, and *pfcrt* 76 T (Y-F-D-T, 51.4%). The average parasite density in malaria cases declined threefold from low-transmission (2010–2013) to pre-elimination period (2014–2016). Logistic regression models showed that patients with younger age (OR for age = 0.97–0.98, p < 0.001), higher initial parasite density (log_10_-transformed, OR = 1.44, p < 0.001), and receiving quinine treatment (compared to artemisinin-based combination therapy, OR = 1.91–1.96, p < 0.001) were more likely to experience treatment failures during follow-up.

**Conclusions:**

*Plasmodium falciparum* in STP had experienced changes in prevalent strains, and increased mutation frequencies in drug-resistance genes from the low-transmission to the pre-elimination settings. Notably, patients with younger age and receiving quinine treatment were more likely to show parasitological treatment failure during follow-up. Therapeutic efficacy should be carefully monitored to inform future treatment policy in STP.

**Supplementary Information:**

The online version contains supplementary material available at 10.1186/s12936-021-04007-3.

## Background

Although global malaria mortality fell by 60% over 2000 to 2019, the progress has leveled off in recent years, with sub-Saharan Africa bearing the highest burden of the disease [1]. High-transmission countries aim at getting back on track to reduce mortality and morbidity, while low-transmission countries aim at eliminating malaria by sustaining the control effort and preventing a rebound in transmission [2, 3]. São Tomé and Príncipe (STP), an island nation located in Central West Africa, has made significant progress toward a low-transmission country through effective vector control interventions [4, 5]. Malaria elimination in STP is promising with the benefits of the relatively isolated location, small population, and single vector and parasite species responsible for malaria transmission [4, 6]. However, STP's progress towards malaria elimination is being threatened by a potential rebound in malaria cases, emergence of insecticide resistant vectors and increased human mobility [4, 7]. In response to this situation, the Taiwan Anti-Malaria Advisory Mission has partnered with the government of STP to reinforce case follow-up by establishing a real-time electronic case management system, and preserving residual dried blood spots (DBSs) from malaria patients for implementation research. By integrating case surveillance data and parasite’s genetic information, the dynamic changes of parasites over the control period can be tracked, mainly focusing on the genetic diversity, anti-malarial drug resistance, and treatment effectiveness in *Plasmodium falciparum*.

The genetic structure of *P. falciparum* in STP was studied before 2004 by analysing the diversity levels of microsatellite loci [8]. The authors detected differences in parasite populations across 1997, 2000, and 2004, showing that local malaria control strategies could cause dynamic changes in parasite populations [8]. Over a decade later, with the expansion and transformation of the national malaria control program, changes in the parasite populations were expected but have not yet been proven. Therefore, this study tracked the genetic diversity of parasites using the merozoite surface protein 1 and 2 (*msp1* and *msp2*). MSP1 and MSP2 are antigens targeted by host-immune responses during blood-stage invasion [9, 10], and are polymorphic markers for identifying genetically distinct parasite subpopulations [11, 12]. MSP1 can be divided into three allelic types, K1, MAD20, and RO33, based on the variable sequences in the block 2 region [13]. MSP2 can be grouped into two dimorphic families, FC27 and 3D7/IC, with different repetitive patterns in the block 3 region [14]. The genetic structure of parasite populations over time can be traced by genotyping these markers in this longitudinal study.

Malaria is diagnosed through passive case detection by microscopy in hospitals and district health centers and through mass screening by rapid diagnostic tests (RDTs, immunochromatographic malaria combo cassette test) [7]. A 3-day course of artesunate-amodiaquine (ASAQ, first-line drug) was given to uncomplicated outpatients, and intravenous quinine was given to severe malaria cases and pregnant women during their first trimester, according to local regulations until 2018 [7, 15]. To follow-up the treatment outcomes, local healthcare workers would visit patients on days 3, 7, 14, 21, and 28 after treatment to collect blood specimens, making blood smears for microscopic examination, and dried blood spots (DBSs) for other research analysis. The second-line drug, artemether-lumefantrine (AL, Coartem®, Novartis), was given to patients with remaining parasites at the follow-up day after the initial treatment [7, 16]. Primaquine was given to patients showing gametocytes during follow-up. A small proportion of recurrent infections observed in STP raised the concerns of possible drug resistance in the parasites. Artemisinin resistance is known to be associated with mutations in the propeller domain of kelch 13 (*pfk13*) [17]. Resistance to ACT partner drugs, including amodiaquine (AQ) and lumefantrine (LF) used in STP, is associated with gene mutations in the multi-drug resistance I (*pfmdr1*) and chloroquine resistance transporter (*pfcrt*) [18, 19]. Thus, this study genotyped these markers coupled with case follow-up data to screen the drug-resistance markers and investigate risk factors for treatment failures in STP.

## Methods

### Ethics statement

The transfer, shipment, and utilization of DBSs and encrypted case surveillance data for research analysis in Taiwan were approved by both the Centro Nacional de Endemias (CNE) in STP (OF^o^ N^o^20/P^o^CNE/2016) and the Research Ethics Committee of National Taiwan University Hospital in Taiwan (NTUHREC No. 201110023RD).

### Study site

**Fig. 1:**
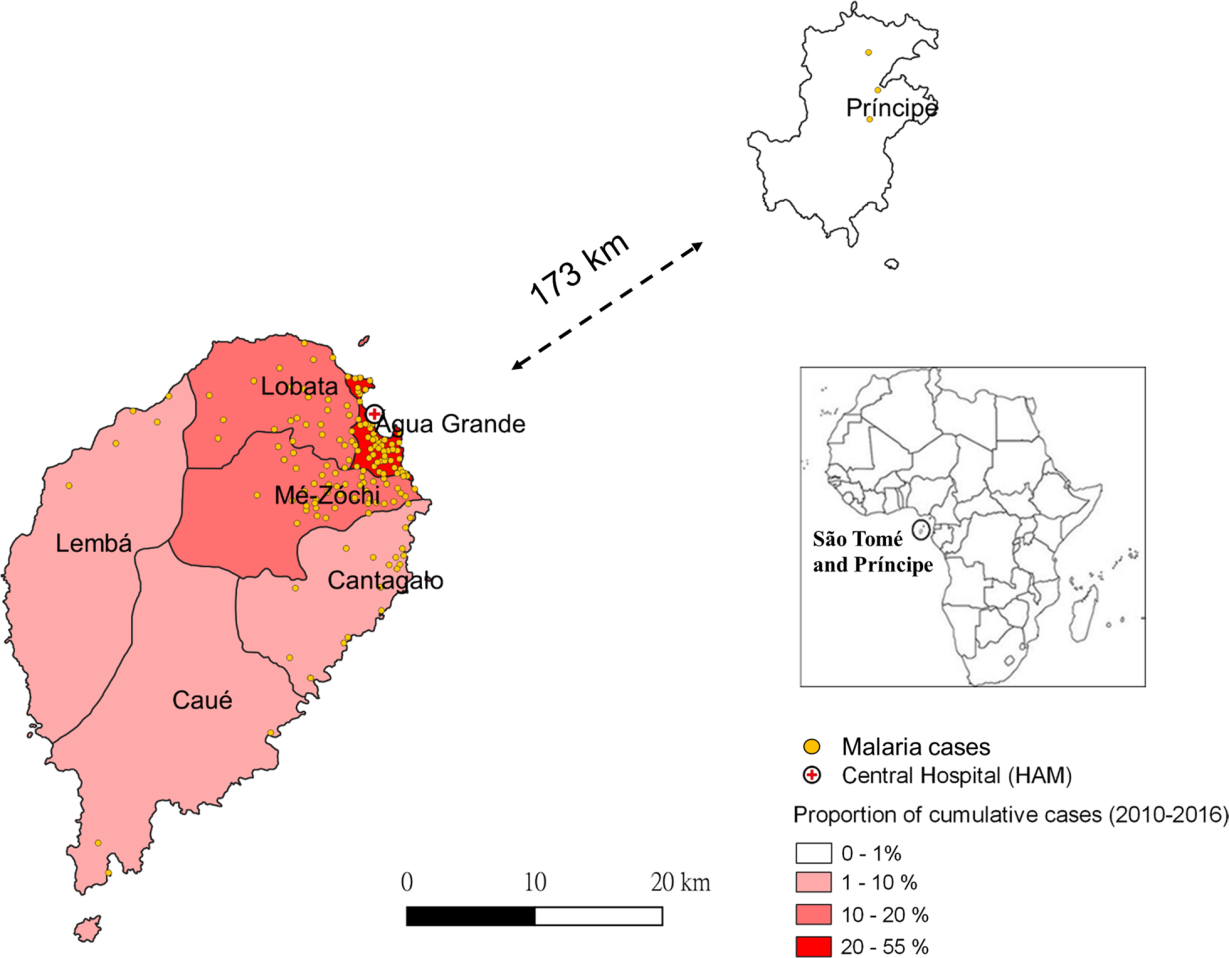
Distribution and proportion of malaria cases from 2010 to 2016 in STP. São Tomé and Príncipe is located in the Gulf of Guinea, central west Africa. São Tomé main island consists of six administrative districts, Água Grande, Mé-Zóchi, Lobata, Cantagalo, Lembá, and Caué. Príncipe offshore island is 173 km away from the São Tomé island. Malaria hotspots mainly distribute at the capital district, Água Grande, and its surrounding districts

STP is located in the Gulf of Guinea, approximately 300 km off the coast of Gabon [20]. The total area is 1001 km^2^ with nearly 200,000 residents [4]. São Tomé main island consists of six administrative districts, Água Grande, Mé-Zóchi, Lobata, Cantagalo, Lembá, and Caué (Fig. 1). Príncipe offshore island is 173 km away from the São Tomé island. The population is primarily concentrated in the plains along the east coast of São Tomé island [4]. Rainfall is frequent throughout the year, except for the dry season from June to early September [4].

### Genomic DNA extraction from dried blood spots (DBSs)

The DBSs were collected from patients who had visited the Central Hospital Ayres de Menezes (HAM) located in the capital district, Água Grande, from 2010 to 2016. This study analyzed two sample sets. First, *msp1* and *msp2* were sequenced in 118 DBSs (day-0, pre-treatment) to identify the infecting parasite strains. In the second sample set, 92 matched samples were from 41 recurrent infections, which contained 41 pre-treatment (day-0) and 51 post-treatment samples (day-R, R = 3, 7, 14, 21, 28, the follow-up days tested positive after treatment). Among the 41 recurrent infections, 36 had one day-R sample, and 5 had more than one day-R samples because they showed parasitaemia on multiple follow-up days. Additionally, 19 pre-treatment samples with negative infections in post-treatment samples were also genotyped for drug-resistance markers. Therefore, the total number of DBSs for drug-resistance genotyping was 111, including 60 pre-treatment (day-0) and 51 post-treatment (day-R) samples.

Genomic DNA was extracted from three 6-mm circular punches on DBSs using the Geneaid Genomic DNA Mini Kit for Tissue (Geneaid Biotech Ltd., Taipei, Taiwan). The blood spots were immersed in 1 mL of lysis buffer and protease K mixture followed by shaking and incubation at 60 ℃ for 1.5 h. The liquid mixture was then transferred to a DNA-binding membrane and washed according to the manufacturer’s instructions. Genomic DNA was eluted in 80 μL of elution buffer and stored at – 20 ℃ for further analysis.

### PCR and sequencing of *msp1* and *msp2*

Block 2 of *msp1* and block 3 of *msp2* sequences were amplified using primer pairs designed in Wooden et al. [21]. The PCR reactions for *msp1* and *msp2* contained 10 μL of 2X HotStarTaq Master Mix (Qiagen, Hilden, Germany), 10 μM of forward and reverse primers, 3 μL of DNA template, and RNase-free water to a total volume of 20 μL. The PCR condition started with an initial denaturation at 95 °C for 15 min, followed by 40 cycles of denaturation at 95 °C for 30 s, annealing at 50 °C for 30 s, extension at 72 °C for 30 s, and a final extension at 72 °C for 10 min using the Biometra TRIO Thermal Cycler (Analytik Jena AG, Jena, Germany). The fragment lengths of the amplified products varied from 200 to 400 bp in *msp1*, and 400 to 600 bp in *msp2*. The confirmed PCR products were gel-purified using QIAquick® Gel Extraction Kit (Qiagen, Hilden, Germany) and sequenced by Applied Biosystems 3730xl DNA Analyzer (Thermo Fisher Scientific, Waltham, MA, USA). The multiplicity of infection (MOI) of *msp1* and *msp2* genes was calculated by averaging the number of amplified bands (haplotypes) per sample.

In addition, nested PCR (nPCR) of *msp1* and *msp2* were performed to discriminate new infections (due to new infectious mosquito bites) and recrudescence (incomplete clearance of asexual parasites after treatment) in matched recurrent samples (n = 92) using the standard methods designed by WorldWide Antimalarial Resistance Network (WWARN) [22, 23]. The nPCR products were confirmed by capillary electrophoresis using the QIAxcel Advanced System (Qiagen, Hilden, Germany) and gel-purified for sequencing. Recrudescence was determined by at least one allele at each locus that was identical between the matched pre and post-treatment samples. New infection was defined when all alleles identified from the post-treatment samples were different from the pre-treatment samples [24].

### Genotyping of polymorphisms on *pfmdr1*, *pfcrt*, and *pfk13*

The *pfmdr1* N86Y, Y184F, D1246Y, *pfcrt* K76T, and *pfk13* gene polymorphisms were analyzed in a total of 60 pre-treatment and 51 post-treatment samples. PCR sequencing was performed to detect *pfmdr1* N86Y, Y184F, and *pfk13* gene polymorphisms [25, 26]. PCR reactions consisted of 10 μL of 2X HotStarTaq Master Mix, 10 μM of forward and reverse primers, 3 μL of DNA template, and RNase-free water to a total volume of 20 μL. The PCR program started with an initial denaturation at 95 °C for 15 min followed by 40 cycles of denaturation at 95 °C for 30 s, annealing at 50 °C for *pfmdr1* and 54 °C for *pfk13*, extension at 72 °C for 30 s, and a final extension at 72 °C for 10 min. The amplicon sizes were 353 bp and 986 bp for the *pfmdr1* and *pfk13* genes, respectively. PCR products were sequenced after confirming sizes by gel electrophoresis.

The *pfmdr1* D1246Y and *pfcrt* K76T alleles were identified by PCR–RFLP. PCR products of *pfmdr1* were digested by EcoRV (20,000 units/mL, New England Biolabs, Massachusetts, USA), and *pfcrt* fragments were digested by ApoI endonuclease (10,000 units/mL, New England Biolabs, Massachusetts, USA) [27, 28]. After the digestion, DNA patterns were visualized on 2.5% agarose gel to determine the polymorphic types. In addition, a subset of the *pfcrt* products was further sequenced to identify the *pfcrt* 72–76 haplotype. Genomic DNA of the 3D7 clone (Catalog Number PRA-405D, ATCC, Virginia, USA) was the wild-type control for the genotyping results of *pfmdr1*, *pfcrt*, and *pfk13* genes.

### Sequence alignment and data analysis

DNA sequences of *msp1* and *msp2* were quality-checked, translated into protein sequences, and aligned based on their structures [29] using Lasergene SeqMan version 7.1 and BioEdit version 7.0. The maximum likelihood (ML) tree was constructed by bootstrapping 100 times using the general time reversible (GTR) model in the MEGA version 7.0. Comparisons of drug-resistance alleles between matched pre-treatment (day-0) and post-treatment (day-R) samples were examined by McNemar’s test. Mixed alleles were treated as mutants in McNemar’s test.

### Characterization of parasite density levels and treatment outcomes of malaria cases in STP

From 2010 to 2016, 10,019 cases were reported by the Central Hospital Ayres de Menezes (HAM). However, only 7482 subjects with complete records were enrolled in the analysis (excluding 2537 cases with incomplete baseline information and those lost to follow-up). The case surveillance data documented patient’s record number, gender, age, residency (district and village), date of confirmed diagnosis, microscopic results before the initial treatment (day-0), treatment regime, and microscopic results at follow-up (days 3, 7, 14, 21, and 28 post-treatment). The parasite density (parasite counts per μL blood) from microscopic results was log_10_-transformed. The linear regression model was used to characterize the significant factors associated with the levels of parasite density.

**Fig. 2:**
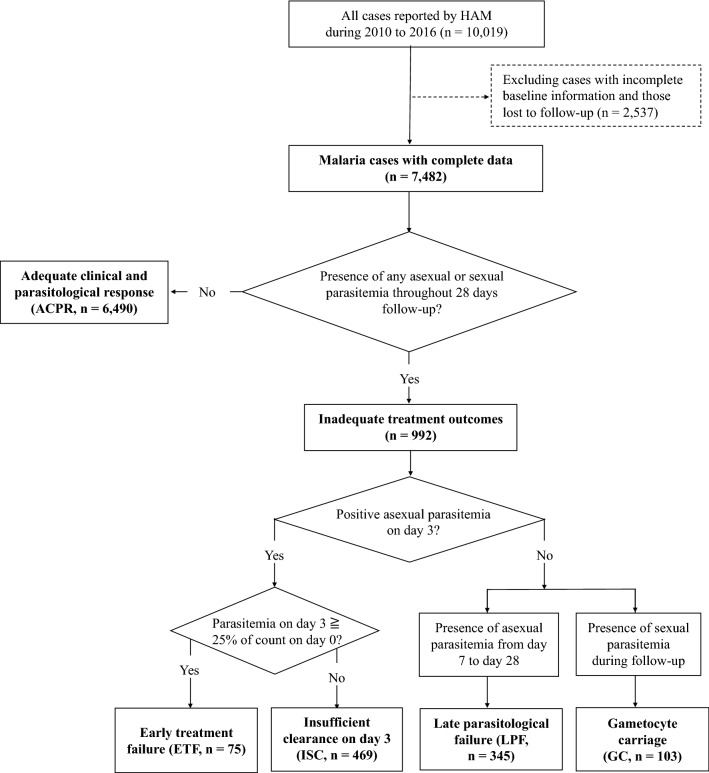
Classification scheme of treatment outcomes in malaria cases reported by HAM. A total of 7,482 cases with complete records are enrolled in the analysis. Cases with inadequate treatment outcomes are further classified into four groups: early treatment failure (ETF), insufficient clearance on day 3 (ISC), late parasitological failure (LPF), and gametocyte carriage (GC) in this study

All patients enrolled in the analysis were classified into one of the following groups: adequate clinical and parasitological response (ACPR), early treatment failure (ETF), insufficient clearance on day 3 (ISC), late parasitological failure (LPF), and gametocyte carriage (GC), as shown in Fig. 2. This classification scheme was modified from the WHO guideline [24, 30]. ISC and GC were the two groups not specified in the WHO guideline, while other groups followed the WHO’s definition [30]. Since recurrent malaria was attributed to the recurrence of asexual parasitaemia [31]; patients showing only sexual parasitaemia (gametocytes) after treatment were separately classified as the gametocyte carriage (GC) group in this study. Early treatment failure (ETF) was defined as the presence of danger signs or higher parasitaemia on day 3, i.e., ≥ 25% of the parasite counts on day-0 [24]. Therefore, those who showed mild symptoms and decreased parasite counts on day 3 were classified into the insufficient clearance (ISC) group in this study.

**Fig. 3:**
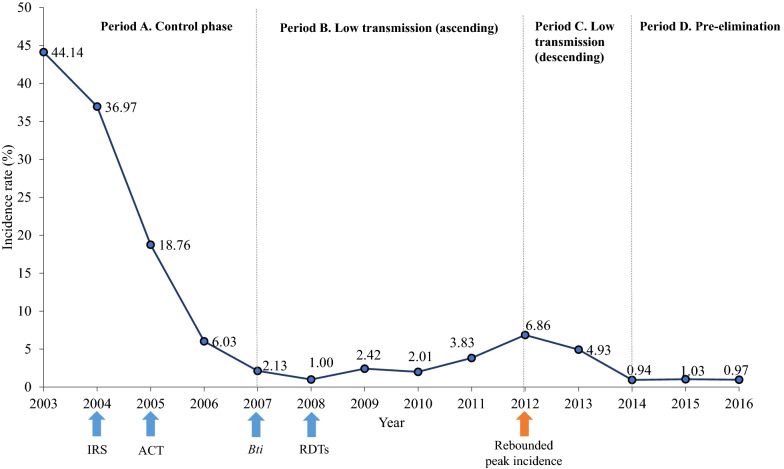
Classification of periods based on the annual malaria incidence rate in STP. Malaria incidence had dropped by 40% during the control phase from 2000 to 2007 due to the prompt deployment of indoor residual spraying (IRS), and the introduction of artemisinin-based combination therapy (ACT). Between 2007 and 2012, the incidence rate was controlled under 5% by applying *Bacillus thuringiensis israelensis* (*Bti*) for larval control, and rapid diagnostic tests (RDTs) for mass screening. Subsequently, there was a slight increase in the incidence, and the rebounded peak was shown in 2012. After the peak incidence, malaria incidence was again controlled to ~1% and reaching the pre-elimination phase

Finally, multivariate logistic regression models evaluated factors associated with early (day 3 positive) and late (day 7–28 positive) parasitological failures after treatment. The host-related variables were the patient’s age, gender (female as reference), and residency (urban districts included Água Grande, Mé-Zóchi, and Lobata compared to other districts). Time-related variables considered the periods and seasons of the initial infections (the year was transformed to “period” as defined in Fig. 3, and the month was transformed to “endemic season” as defined in Additional file 1: Fig. S1). Treatment outcomes of ACT (reference) and quinine groups were compared in the logistic regression models. Mediation analysis was performed to clarify the relationship between the initial parasite density, treatment types, and parasitological failures. All statistical analyses were performed by R version 4.0.2.

## Results

### Sequence diversity of *msp1* block 2 in *P. falciparum* isolates from STP

Twenty-two haplotypes were identified in *msp1* block 2 from 118 samples collected between 2010 and 2016 (GenBank accession numbers MW001371–MW001392). Nine haplotypes belonged to the K1 family (KH1–KH9), nine belonged to the MAD20 family (MH1–MH8, haplotype MH3 was translated from two nucleotide sequences, MH3-1 and MH3-2), and four belonged to the RO33 family (RH1–RH4). The majority of cases (93.2%, 110/118) were infected by a single clone of *msp1*, with only 6.8% (8/118) of cases showing multi-clones of *msp1* haplotypes (mean MOI = 1.08). K1, MAD20, and RO33 haplotypes were identified in 34.7% (41/118), 44.1% (52/118), and 21.2% (25/118) of the total isolates, respectively.

The K1 and MAD20 alleles shaped the diversity by rearranging a number of tri-peptide repeat units (the R1 region of Additional file 1: Fig. S2). According to the repetitive patterns and arrangement, two subgroups were further divided in the K1 and MAD20 families on the phylogenetic tree (Additional file 1: Fig. S3). One group contained haplotypes that were more widespread relative to the other group. On the other hand, the RO33 haplotypes were unique sequences without any repeats and presented the least polymorphisms in the amplified region.

**Fig. 4:**
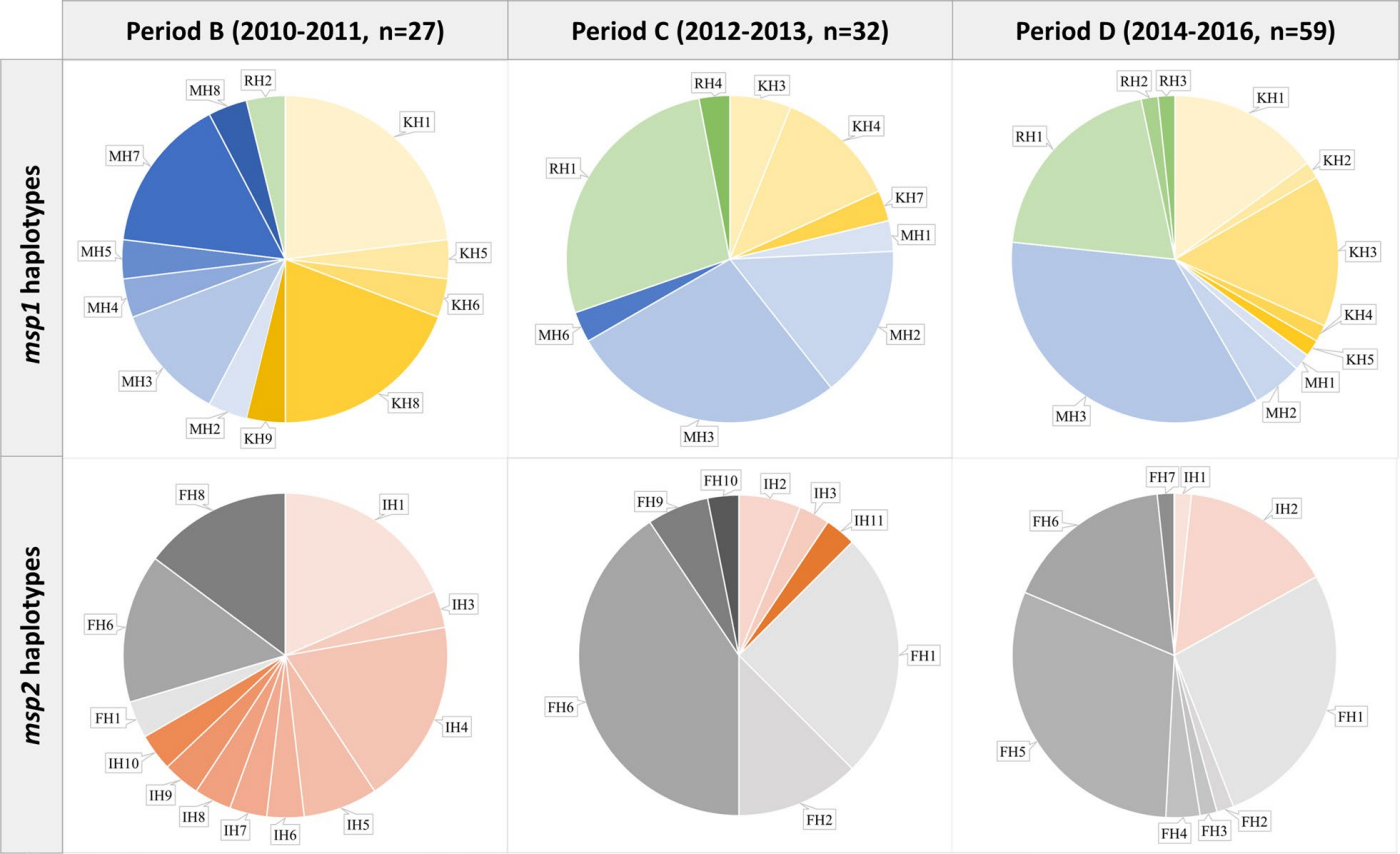
Distribution of *msp1* and *msp2* haplotypes in *P. falciparum* isolates from 2010 to 2016 in STP. The pie charts show the distribution of *msp1* and *msp2* haplotypes isolated from parasites across three different periods in STP. Period B and C are the low transmission periods with ascending and descending trend, respectively. Period D is the pre-elimination phase. The *msp1* haplotypes include KH1-KH9 (yellow colors, K1 family), MH1-MH8 (blue colors, MAD20 family), and RH1-RH4 (green colors, RO33 family). In *msp2*, 11 haplotypes are found in the 3D7/IC family (IH1-IH11, orange colors), and 10 haplotypes are found in the FC27 family (FH1-FH10, gray colors)

Allele distribution of *msp1* had changed during, and after the peak incidence in 2012 (χ^2^ = 7.94, df = 2, p = 0.02) that prevalence of K1 alleles decreased from 54 to 35%; prevalence of MAD20 ranged from 42 to 49%, and that of RO33 alleles increased from 4 to 23% (Fig. 4). The increase of MAD20 and RO33 alleles was mainly due to the increased prevalence of haplotypes MH3 and RH1, for more than 20% growth after 2012, and replaced K1 haplotypes becoming the predominant types after the peak incidence.

### Sequence diversity of *msp2* block 3 in *P. falciparum* isolates from STP

Twenty-one haplotypes were identified in 118 *msp2* sequences, of which 11 belonged to the 3D7/IC family (IH1–IH11, GenBank accession numbers MW001393–MW001403), and 10 belonged to the FC27 family (FH1-FH10, GenBank accession numbers MW001404–MW001413). Nearly all the samples (98.3%, 116/118) were infected by a single clone of *msp2*, with only 1.7% (2/118) presenting multi-clones of *msp2* (mean MOI = 1.02). The 3D7/IC and FC27 alleles were detected in 27.1% (32/118) and 72.9% (86/118) of the total isolates, respectively.

Two tandem repeat regions (R1 and R2) were flanked by three family-specific regions (E1–E3, Additional file 1: Fig. S4) in *msp2* block 3. The R1 region in 3D7/IC haplotypes consisted of GSA-rich repeats in differed lengths, resulting in diverse polymorphisms. The R2 region of 3D7/IC was composed of 5–14 poly-threonine (T) residues, and only haplotypes IH2 and IH9 presented additional copies of the TPA motif before the poly-threonine segments. Genetic diversity of the 3D7/IC alleles was the highest in 2010–2011 (9 haplotypes being detected) and then decreased to only 1–3 haplotypes with low prevalence (9–17%) afterward (Fig. 4).

The FC27 haplotypes could be divided into two subgroups. One group included FH1, 2, and 3, containing three copies of the family-specific repeats (32 amino acids) in the R1 region, without showing tandem repeats in the R2 region. In contrast, the other group comprised FH4 to FH10, showing tandem repeats of 12 amino acids (aa) in the R2 region, without presenting any repeats in the R1 region (Additional file 1: Fig. S4). Among the detected FC27 types, haplotypes FH1, FH5, and FH6 were the dominant types with frequencies of 29.1% (25/86, detected in all periods), 20.9% (18/86, detected only in 2014–2016), and 31.4% (27/86, detected in all periods), respectively (Fig. 4).

Similar to *msp1*, the allele distribution of *msp2* varied across different periods (Fig. 4). The 3D7/IC allelic family was predominant with 66.7% (18/27) of the prevalence before 2012; however, the prevalence declined by 50% and became minor alleles after 2012. Instead, the FC27 alleles became the predominant types, accounting for 85%-88% of the prevalence during and after the peak incidence.

### Prevalence of anti-malarial drug resistance mutations in *P. falciparum* isolates from STP

Anti-malarial drug resistance markers were genotyped in 60 pre-treatment samples and 51 post-treatment samples collected from 2014 to 2016. The dominant alleles were *pfmdr1* 86Y (82.9%, 92/111), *pfmdr1* 184F (62.2%, 69/111), *pfmdr1* D1246 (96.4%, 107/111), and *pfcrt* 76 T (92.8%, 103/111) (Table 1). Thus, the dominant allelic types of *pfmdr1* 86, 184, 1246-*pfcrt* 76 were YFD-T (51.4%, 57/111), followed by YYD-T (27.0%, 30/111). No mutations were found in the *pfK13* sequences.

**Table 1:** Antimalarial drug-resistance mutations in the pre-treatment and post-treatment samples

Antimalarial drug-resistance markers	Pre-treatment (n = 60)	Post-treatment (n = 51)	Total (n = 111)
	No. (%)	No. (%)	No. (%)
*pfmdr1* 86			
N	8 (13.3)	10 (19.6)	18 (16.2)
Y	52 (86.7)	14 (78.4)	92 (82.9)
N/Y	0 (0.0)	1 (2.0)	1 (0.9)
*pfmdr1* 184			
F	41 (68.3)	28 (54.9)	69 (62.2)
Y	19 (31.7)	23 (45.1)	42 (37.8)
*pfmdr1* 1246			
D	58 (96.7)	49 (96.1)	107 (96.4)
Y	2 (3.3)	2 (3.9)	4 (3.6)
*pfcrt* 76			
K	1 (1.7)	5 (9.8)	6 (5.4)
T	59 (98.3)	44 (86.3)	103 (92.8)
K/T	0 (0.0)	2 (3.9)	2 (1.8)
*pfmdr1* 86 + 184 + 1246-*pfcrt* 76			
YFD-T	33 (55.0)	24 (47.1)	57 (51.4)
YYD-T	16 (26.7)	14 (27.5)	30 (27.0)
NFD-T	6 (10.0)	3 (5.9)	9 (8.1)
YFD-K	2 (3.3)	1 (2.0)	3 (2.7)
NYD-K	0 (0.0)	3 (5.9)	3 (2.7)
YYY-T	1 (1.7)	1 (2.0)	2 (1.8)
NYD-K/T	0 (0.0)	2 (3.9)	2 (1.8)
NYD-T	1 (1.7)	1 (2.0)	2 (1.8)
NYY-T	1 (1.7)	1 (2.0)	2 (1.8)
N/YYD-K	0 (0.0)	1 (2.0)	1 (0.9)

The post-treatment samples were from 41 patients showing inadequate treatment outcomes, of which 22% (9/41) showed insufficient clearance of parasites on day 3, 22% (9/41) showed early treatment failure, 31.7% showed late parasitological failure (13/41), and 24.4% (10/41) carried gametocytes during follow-up. Thirty-one (75.6%, 31/41) of these patients were hospitalized and treated with quinine, while 10 (24.4%, 10/41) were uncomplicated cases and treated with ASAQ on day-0. Matched-pair comparisons of *pfmdr1* and *pfcrt* polymorphisms showed no significant differences found between the matched pre and post-treatment samples. The results showed that 83% (34/41) of the recurrent infections shared identical alleles of *msp1*, *msp2*, *pfmdr1*, and *pfcrt* between pre and post-treatment samples, suggesting that the positive parasitaemia during follow-up was due to recrudescence. However, seven patients (17%, 7/41) showed different *pfmdr1* or *pfcrt* patterns between day-R and day-0 (Additional file 1: Table S1). Among these seven patients, five showed substitutions one week after treatment, while two showed early substitutions on day 3. The substitutions mainly changed from *pfmdr1* 86Y to N86, 184F to Y184, *pfcrt* 76 T to K76 after quinine treatment (6 patients), and ASAQ treatment (1 patient) at baseline. One patient treated with AL during follow-up showed substitutions from *pfmdr1* 86Y to N86 and *pfcrt* 76 T to K76 (mutation types against LF) on days 7, 9, and 14. Moreover, nPCR results in these seven patients showed that four were confirmed to have multi-clonal and new infections in the post-treatment samples.

Significant temporal change of *pfmdr1* Y184F was found in the 60 pre-treatment samples from 2014 to 2016 (χ^2^ = 8.25, df = 2, p = 0.02). The *pfmdr1* 184F type showed a significant increase of 39% from 2014 to 2016 (Additional file 1: Fig. S5). The *pfmdr1* 86Y (75–95%) and *pfcrt* 76 T (95–100%), associated with the reduced sensitivity of AQ, were constantly prevalent in the parasite population. The *pfcrt* 76 T mutation was nearly fixed in the local parasite population. The *pfcrt* 72–76 mutant type was identified as the CVIET haplotype from sequencing results.

### Characteristics of parasite density levels, treatment outcomes, and factors associated with parasitological recurrence

A total of 7482 cases were analysed in the regression models (Table 2), and results showed that parasite density levels significantly decreased following the increase of age (coefficient = − 0.012, p < 0.001). Parasite density in the infective hosts was significantly higher (coefficient = 0.4, p < 0.001) in the low-transmission period (average log_10_ parasite density = 3.4) than in the pre-elimination phase (average log_10_ parasite density = 2.9), approximately 3.2-fold difference in the average counts of parasites in blood.

**Table 2:** Factors correlated with log_10_ parasite density (no. of observations = 7482)

Variables	Coefficient	SE	z value	p-value
Age	− 0.012	0.001	− 17.801	< *0.001*
Male	0.011	0.022	0.516	0.606
Endemic season	0.033	0.022	1.474	0.141
Period B vs. D	0.393	0.031	12.490	< *0.001*
Period C vs. D	0.404	0.028	14.319	< *0.001*
Urban	− 0.139	0.070	− 1.984	0.050

Factors correlated with the levels of parasite density (log_10_ transformed) are characterized using linear regression model. The definition of each variable is described in the method section. Parasite density in the low transmission periods (Period B and C, 2010–2013) are compared with that in the pre-elimination phase (Period D, 2014–2016) using dummy variables. Significant p-values (p < 0.05) are shown in italic

**Fig. 5:**
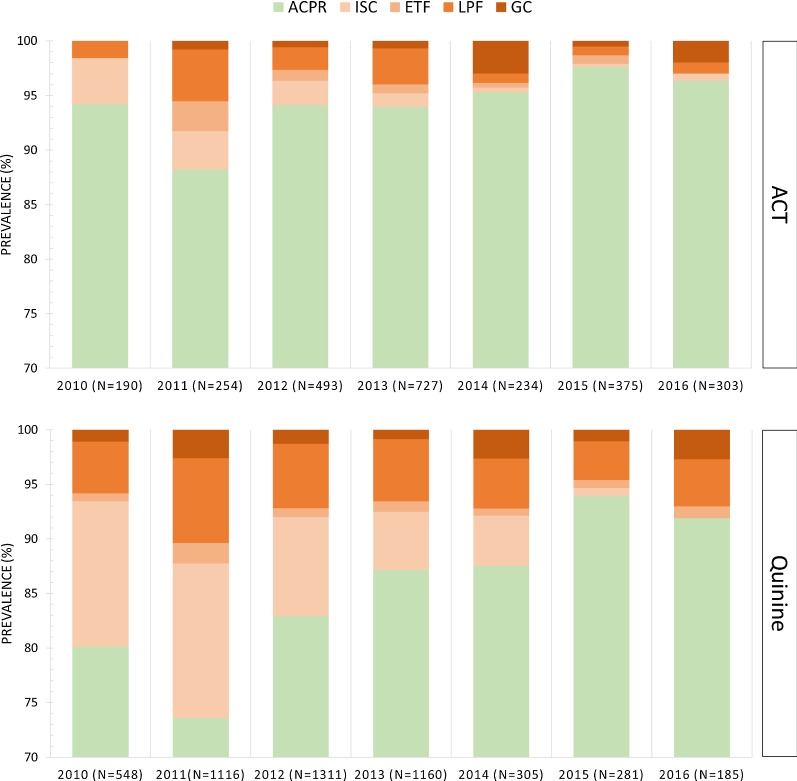
Treatment outcomes of ACT and quinine from 2010 to 2016 in STP

The adequate clinical and parasitological responses (ACPR) rate was 86.7% (6490/7482) among total cases (Fig. 5). ACPR rate in the ACT treatment group (88.2–97.6%) was slightly higher than that in the quinine treatment group (73.6–94.0%). The proportion of cases with inadequate treatment outcomes in the quinine group (17.3%, 847/4906) was higher than that in the ACT group (5.6%, 145/2576). In particular, patients treated with quinine were mainly hospitalized patients with higher parasitaemia levels at enrollment, and their follow-up assessments showed higher rates of insufficient clearance on day 3 (0.7–14.2% in quinine group; 0.3–4.2% in ACT group), and late parasitological failure after day 7 (3.6–7.8% in quinine group; 0.8–4.7% in ACT group). The proportion of cases treated by quinine (61.5–81.5%) was higher than that treated by ACT (18.5–38.5%) in the low-transmission period. Following the decrease of infective parasite density in the pre-elimination settings, cases treated by ACT were greater or nearly equal to the quinine-treated numbers, and the overall ACPR rate was higher in the pre-elimination phase (94%) than in the low-transmission period (84.6%).

Logistic regression models were constructed to characterize risk factors for early and late parasitological treatment failures during follow-up (Table 3). Results showed that age was a significant protective factor against parasitological treatment failures (OR = 0.97 and 0.98, p < 0.001 for early and late parasitological failure, respectively). Thus, younger children had higher odds of recurrence than the elderly. In the low-transmission period, the odds of early and late parasitological failures were significantly higher than those in the pre-elimination period (OR = 1.63–5.82, p < 0.01). Both parasite density (log_10_ transformed, OR = 1.44, p < 0.001) and quinine treatment (OR = 1.96, p < 0.001) had significant association with early parasitological failure. However, only quinine treatment (OR = 1.91, p < 0.001) had significant association with late parasitological failure, and parasite density had no significant association with late parasitological failure after adjusting treatment types (p = 0.27, Additional file 1: Fig. S6).

**Table 3:** Logistic regression analyses of factors related to early and late parasitological failures (no. of observations = 7482)

Variable	Coefficient	SE	z value	p-value	OR (95% CI)
*Early parasitological failure* *(day 3 positive)*					
Age	− 0.028	0.004	− 6.752	< *0.001*	0.97 (0.96**–**0.98)
Male	− 0.048	0.093	− 0.513	0.608	0.95 (0.80**–**1.14)
Urban	0.015	0.254	0.059	0.953	1.02 (0.62**–**1.67)
Period B vs. D	1.761	0.199	8.860	< *0.001*	5.82 (3.94**–**8.59)
Period C vs. D	1.003	0.199	5.046	< *0.001*	2.73 (1.85**–**4.03)
Endemic season	0.026	0.093	0.278	0.781	1.03 (0.86**–**1.23)
Log_10_ parasite density at day 0*	0.368	0.058	6.379	< *0.001*	1.44 (1.29**–**1.62)
Quinine*	0.672	0.150	4.490	< *0.001*	1.96 (1.46**–**2.63)
*Late parasitological failure* *(days 7–28 positive)*					
Age	− 0.022	0.005	− 4.775	< *0.001*	0.98 (0.97**–**0.99)
Male	− 0.173	0.112	− 1.542	0.123	0.84 (0.67**–**1.05)
Urban	− 0.239	0.296	− 0.807	0.419	0.79 (0.94**–**1.22)
Period B vs. D	0.647	0.189	3.419	< *0.001*	1.91 (1.32**–**2.77)
Period C vs. D	0.487	0.181	2.691	*0.007*	1.63 (1.14**–**2.32)
Endemic season	− 0.089	0.113	− 0.788	0.430	0.91 (0.73**–**1.14)
Log_10_ parasite density at day 0*	0.069	0.064	1.071	0.284	1.07 (0.94**–**1.22)
Quinine*	0.648	0.162	3.997	< *0.001*	1.91 (1.39**–**2.63)

The definition of each variable is described in the method section. Period B and C are the low transmission periods with ascending and descending trend, respectively. Period D is the pre-elimination phase. The asterisk denotes that the mediation analysis was performed to examine the relationship between “parasite density → treatment → early/late parasitological failures” in Additional file 1: Fig. S6. Significant p-values (p < 0.05) are shown in italic

## Discussion

This study tracked the dynamic changes in genetic diversity, anti-malarial drug resistance of parasites, and treatment follow-up of malaria cases in STP. The prevalent parasite strains changed during the transition time from the low-transmission to the pre-elimination settings. Moreover, patients with younger age, higher parasite density at enrollment, and receiving quinine treatment were more likely to experience recurrence during follow-up, which was majorly due to recrudescent infections based on the genotyping results.

A previous study analysed 180 *P. falciparum* isolates from STP in 2000 and showed that the frequency of *msp1* K1, MAD20, and RO33 types was 50%, 44%, and 6%, respectively [12]. This was similar to the frequency detected in the samples from 2010 to 2011 (K1 = 54%, MAD20 = 42%, RO33 = 4%) in this study. After the peak incidence in 2012, the frequencies of the MAD20 and RO33 allelic types increased and replaced K1 types as the prevalent alleles. Similar results were also found in *msp2*. The proportion of *msp2* 3D7/IC and FC27 alleles detected from 2010 to 2011 was similar to that in 2000 [12], of which 3D7/IC was the prevalent allelic type (60–65%), and FC27 was the minor type (35–40%). However, after 2012, FC27 replaced 3D7/IC as the dominant *msp2* alleles. The changing of genetic makeups could be associated with malaria transmission intensity and was shown in studies from other island countries [32, 33]. For example, a generally high parasite genetic variation in *msp1* and *msp2* was found after deploying multiple malaria control measures since 2004 on Bioko Island, the neighboring island of STP [32]. A progressive decrease in parasite genetic diversity was observed due to the declined malaria transmission intensity after the introduction of ACT on the Grande Comore Island, located off the southeast coast of Africa [33]. Generally, STP was under low transmission intensity, and the mean MOI was low across the study period. Still, changes of *msp1* and *msp2* compositions in parasites were detected and coincident with the transmission trend. Although with limited sample numbers, the reasons for this changing deserved further discussion and investigation.

The haplotypes that showed expansion after 2012 in STP were *msp1* MH3 and RH1, and *msp2* FH5 and FH6. The *msp1* MH3 and *msp2* FH6 were found before 2012 and increased in frequencies afterward. The *msp1* RH1 and *msp2* FH5 were not detected in the samples collected before 2012. Based on these findings, this study supposed the changes of the prevalent strains might be due to the population expansion of a few haplotypes that originally existed locally, or some new strains that may have evolved or emerged through the recombination or importation events [34–36]. The expansion of a few parasite clones could be attributed to several factors, including selective antibodies, anti-malarial drug pressures, importation, or a founder effect at the beginning of the epidemics [34, 37, 38]. Changes in the prevalent types before, during, and after the outbreak were also identified in Djibouti, Northern East Africa, and were suggested to be associated with the expansion of a few strains that were already prevalent during the epidemics in 1999 [37]. Another study in Myanmar showed that the population structure of *msp1* and *msp2* had diversified drastically in 2013–2015, compared to the previous years, 2004–2006, and could be due to a higher level of intragenic recombination estimated in the recent population [36]. For islands like STP, human mobility and malaria importation are of great challenges, especially importing from mainland Africa where malaria transmission is more intense [39–41]. Examples from Bioko Island showed that much of the *P. falciparum* parasites currently observed on the island could probably be attributed to imported cases from Equatorial Guinea [40, 41]. Future studies could verify these plausible factors by utilizing more genomic, epidemiological, and mobility data to reveal the rebounded causes and threats in STP.

Anti-malarial drug resistance is another critical challenge to malaria control and elimination [42]. According to the policy decision recommended by the WHO [43], ACT should be changed if the proportion of treated patients remaining parasitaemic on day 3 exceeded 10%. In STP, the proportion of patients showing positive parasitaemia on day 3 after ACT treatment was 2.5% (63/2,576), showing that the current treatment policy was acceptable. However, by monitoring target-site mutations, this study found an increase in the *pfmdr1* 86Y mutation compared to that in a previous study conducted in 2004 when ACT was introduced in STP [44]. The prevalence of the *pfmdr1* 86Y mutation in parasite isolates increased from 21% in 2004 to 87% in 2014–2016. This mutation is associated with the reduced sensitivity to AQ and chloroquine (CQ) in *P. falciparum* [45]. With the increased *pfmdr1* 86Y mutation and nearly fixed mutation of *pfcrt* 76 T, the local parasites may show increased tolerance to AQ, raising concerns regarding the use of AQ as the first-line ACT partner drug in STP. Other polymorphisms showed the same pattern of predominance (*pfmdr1* 184F and *pfmdr1* D1246) as shown in the parasites isolated in 2004 [44]. Overall, the prevalent haplotype of *pfmdr1* and *pfcrt* was YFD (51.4%) and CVIET (92.8%), which was similar to the findings in the neighbouring Bioko island, where the *pfmdr1* YFD (45–59%) and *pfcrt* CVIET (92%) were also the prevalent types from 2011 to 2014 [46, 47].

The drug-resistance genotyping results found that most parasites detected after treatment were recrudescent infections, showing identical genotypes as the initial infections. However, a few recurrent infections (seven patients) showed substitutions of drug resistance types after treatment. The observed substitutions in these seven patients could be owing to two reasons. First, multi-clonal and new infections were detected in four post-treatment samples, showing that they were infected by additional or new parasite strains that carry the alternative alleles. Second, the substitutions of AL resistant types (*pfmdr1* 86Y → N86, and *pfcrt* 76 T → K76) were detected in patients treated by the second-line drug, AL, during follow-up. This suggested possible rapid selection against AL treatment. However, AL was not popularly used in STP compared to ASAQ. Therefore, the drug-resistance selection of AL will require further investigation with larger sample size.

This study found that young children had a higher risk of showing high parasitaemia and treatment failures, probably due to the lack of acquired immuno-protection and lower treatment compliance compared to adults [48]. Notably, patients treated with quinine were more likely to show insufficient clearance of parasites during follow-up than those treated with ACT. Several possible reasons were suggested as follows. First, patients treated with quinine mostly had higher parasitaemia levels, prolonging the clearance time. The mean parasite clearance time of quinine treatment was approximately 4–5 days [49, 50], which was slower than ASAQ treatment (2–3 days) in general [51], and the difference may be more significant in cases with higher parasitaemia. Second, poor compliance and tolerability with the quinine regimen may occur, especially in children. The recommended quinine regimen in sub-Saharan Africa is 10 mg/kg administered three times daily for 7 days [43, 49]. This prolonged treatment course may reduce patients’ compliance if they have to take medicines after being discharged from the hospital. Studies in Africa have found unacceptably high treatment failure rates for patients who did not complete the 7-day quinine regimen [52, 53]. One study in Uganda investigated children administered a 7-day course of oral quinine, and found 69% (18/26) showed recrudescence during follow-up. The primary causes were poor adherence due to the caregivers forgetting to administer the drugs, the drugs being vomited up, or the children feeling better [54]. This was similar to the observations in STP. The study results also proved that most of the recurrent infections in quinine group were due to the incomplete clearance of the initially infected parasites, with only a few due to new infections. The final reason was the possible development of resistance against quinine in the parasite population. The mechanism of quinine resistance has not been well elucidated [53]. Conflicting results from the lack of resistance to varying degrees of resistance against quinine have been reported in Africa [55, 56]. Notably, one in vitro study from Western Kenya showed polymorphisms in *pfmdr1* 86Y, 184F, and *pfcrt* 76 T were significantly associated with reduced quinine susceptibility in *P. falciparum* [57]. This suggests that the parasite strains in STP may have developed reduced sensitivity in quinine owing to the high prevalence of *pfmdr1* 86Y, 184F, and *pfcrt* 76 T detected in this study. However, the contribution of these genetic polymorphisms differed among strains; thus, the association with in vitro quinine susceptibility should be further assessed in the STP isolates [57].

Quinine has been used since chloroquine was abandoned in STP due to high resistance in the 1990s [49]. According to local regulations, it has been used for severe malaria treatment, at least until 2018 [4, 7, 15, 16, 58]. One possible reason why quinine has long been used may be that the medicinal plant for quinine (Cinchona tree) is abundant on Sao Tome and Principe Islands [59], and the communities are more adapted to quinine as one of the most-used anti-malarial drugs. However, following the WHO Guidelines for Treatment of Malaria [43] and the current findings, it was suggested to replace IV quinine with IV artesunate. According to the latest WHO malaria report 2020 [1], the local government has updated their anti-malarial drug policies in 2019, using IV artesunate to treat severe malaria now. Although no mutations were found in the artemisinin resistance marker in this study, the *pfk13* mutations have been reported in a few African parasites recently, for example, the emergence of *pfk13*-mediated artemisinin resistance in Rwanda [60] and Uganda [61], which should be carefully monitored in all the African isolates in the future.

Overall, the increased outdoor mosquito density and pyrethroid resistance [4], the changes of parasites’ antigenic alleles and drug resistance mutations, and a higher treatment failure in young children treated by quinine could be significant challenges for malaria elimination in STP. Moreover, surveillance data showed that malaria hotspots in STP are distributed nearby the Central Hospital (HAM), military camps, schools, markets, and airports located in the capital district, Água Grande. These are places that aggregate many people and may intensify malaria transmission. Although the higher case numbers observed in these hotspots may be due to the accessibility to medical services, poor environmental management in these densely populated locations pose a great threat to the rebound of malaria. Concerning malaria elimination, these specified risk factors and hotspots should be addressed in the policy decision-making for the national malaria control programmes in STP.

## Conclusion

Although malaria treatment efficacy remained acceptable in STP, this study found that the circulating parasites underwent temporal changes of prevalent strains and increased frequency of drug resistance mutations. Case surveillance data showed that patients with younger age, higher parasitaemia level at enrollment, and receiving quinine treatment were more likely to experience recurrence during follow-up. It is recommended that the latest therapeutic efficacy should be monitored, at least in children and in the hotspots, to avoid the rebound in transmission and the spread of drug-resistant parasites.

## Supplementary Information

**Additional file 1: Fig. S1.** Monthly malaria cases in HAM from 2010 to 2016. **Fig. S2.** Sequence alignment of MSP1 haplotypes. **Fig. S3.** Phylogenetic tree of the *msp1* sequences from STP and other countries. **Fig. S4.** Sequence alignment of MSP2 haplotypes. **Fig. S5.** Temporal changes of *pfmdr1* and *pfcrt* polymorphisms in 60 pre-treatment samples from 2014 to 2016. **Fig. S6.** Relationship between initial parasite density, treatment types, and parasitological treatment failures. **Table S1.** Substitutions of *pfmdr1* and *pfcrt* genotypes after treatment in seven recurrent infections.

## Data Availability

All data generated or analyzed in this study are included in this published article and its additional files.
