# Supplementary material for: Dynamic changes in genetic diversity, drug resistance mutations, and treatment outcomes of falciparum malaria from the low-transmission to the pre-elimination phase on the islands of São Tomé and Príncipe
Source: Malar J. 2021 Dec 14;20:467. doi: 10.1186/s12936-021-04007-3 (PMC8672503; doi:10.1186/s12936-021-04007-3)
Supplement: Supplementary file 1 — Additional file 1: Fig. S1. Monthly malaria cases in HAM from 2010 to 2016. Fig. S2. Sequence alignment of MSP1 haplotypes. Fig. S3. Phylogenetic tree of the msp1 sequences from STP and other countries. Fig. S4. Sequence alignment of MSP2 haplotypes. Fig. S5. Temporal changes of pfmdr1 and pfcrt polymorphisms in 60 pre-treatment samples from 2014 to 2016. Fig. S6. Relationship between initial parasite density, treatment types, and parasitological treatment failures. Table S1. Substitutions of pfmdr1 and pfcrt genotypes after treatment in seven recurrent infections. [file 12936_2021_4007_MOESM1_ESM.docx]

**
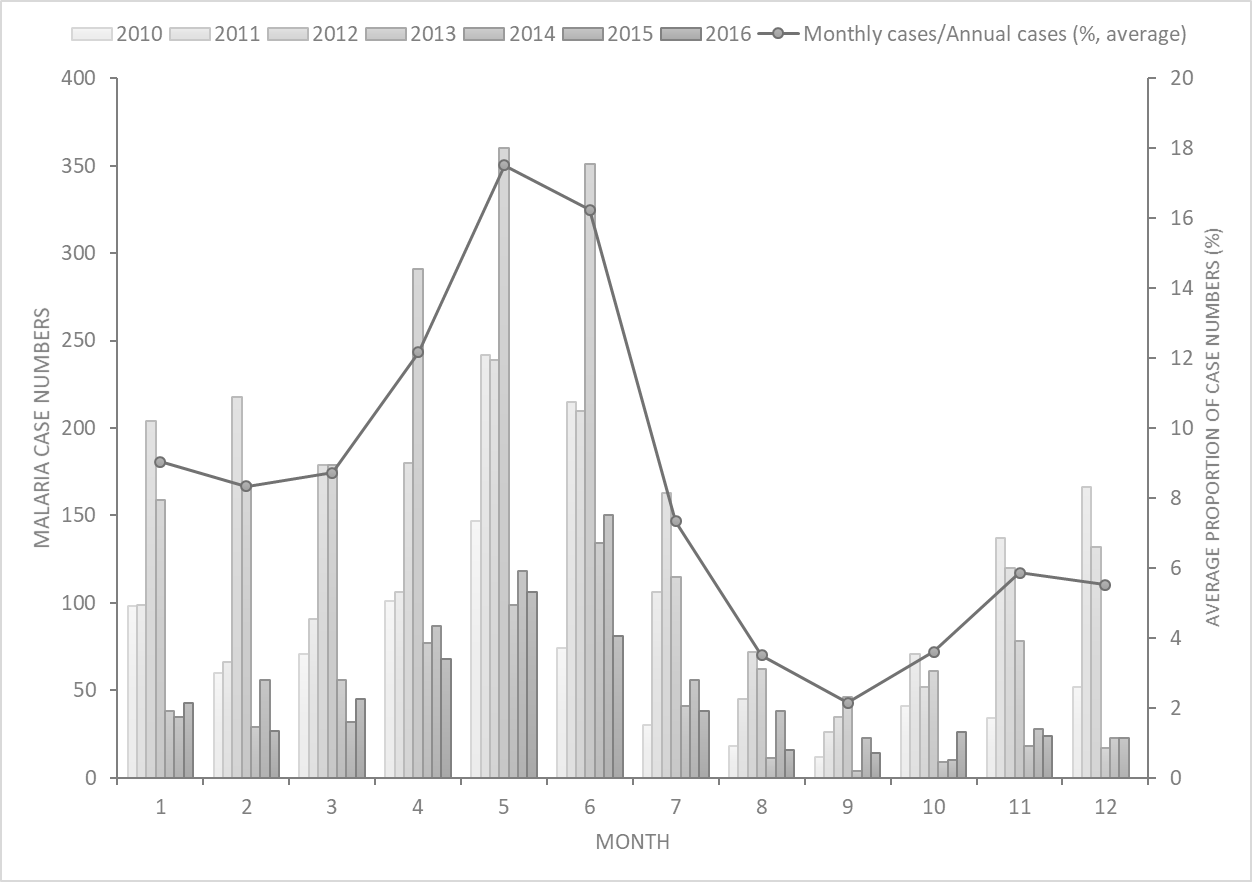
**

**Fig. S1 Monthly malaria cases in HAM from 2010 to 2016.**

The endemic season is defined as the period with average monthly cases that accounted for more than 10% of annual cases in this study, from April to June.

**(A) K1 family (9 haplotypes)**


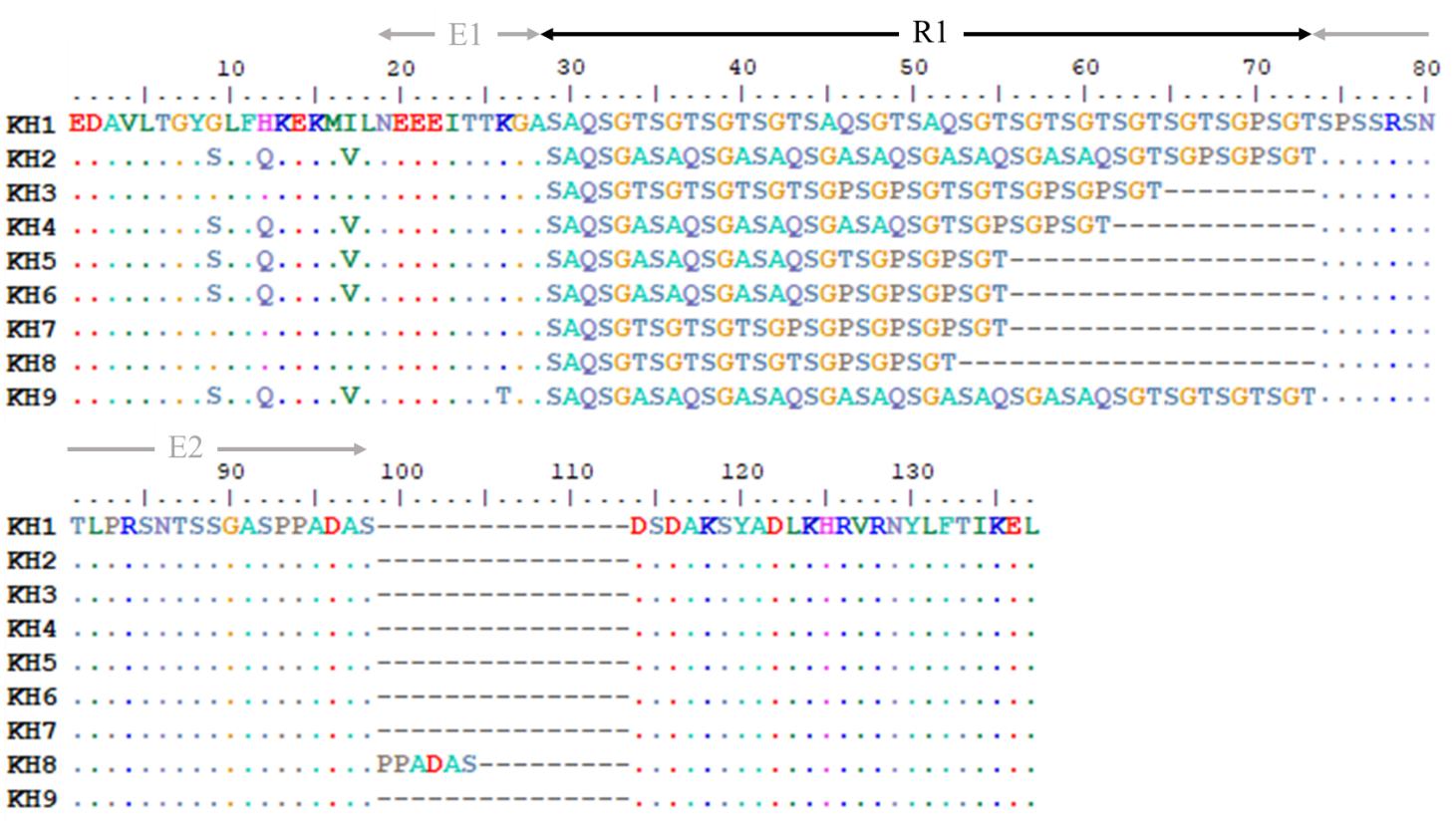


**(B) MAD20 family (8 haplotypes)**


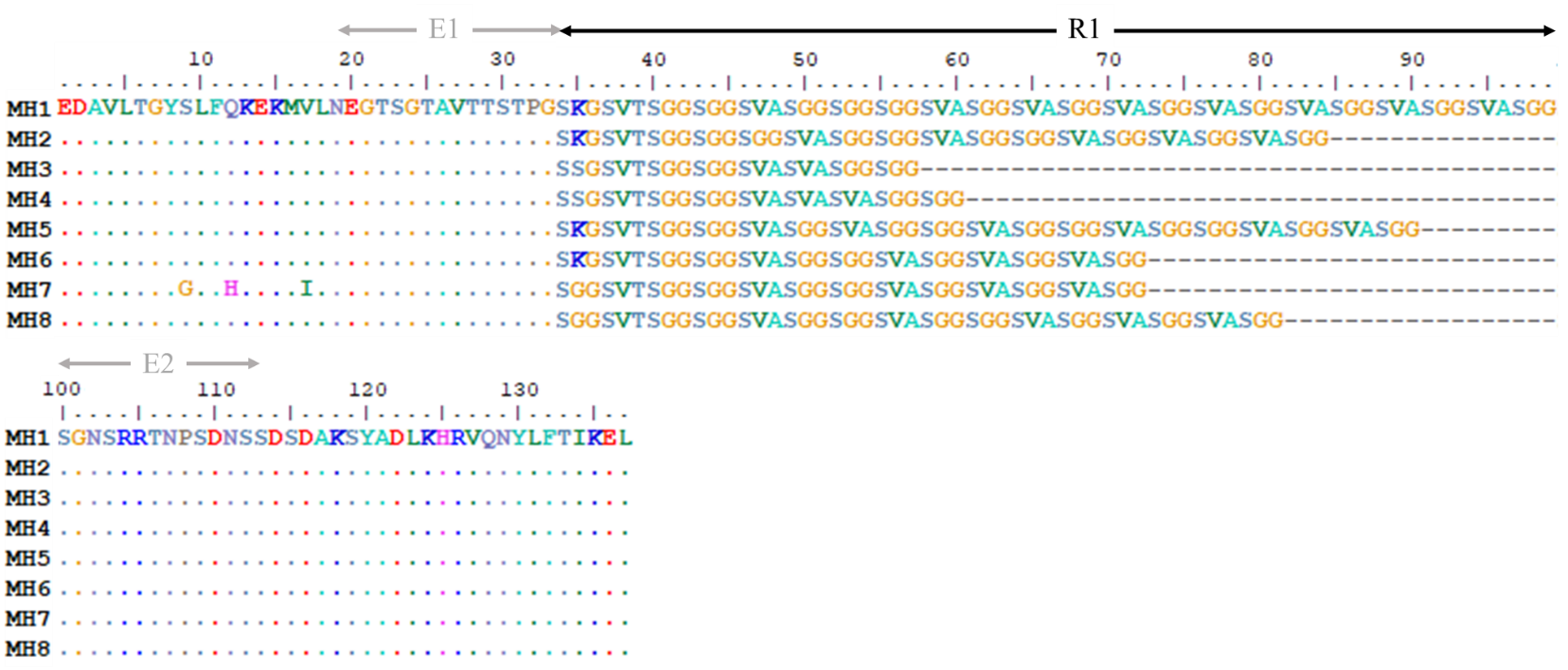


**(C) RO33 family (4 haplotypes)**

**
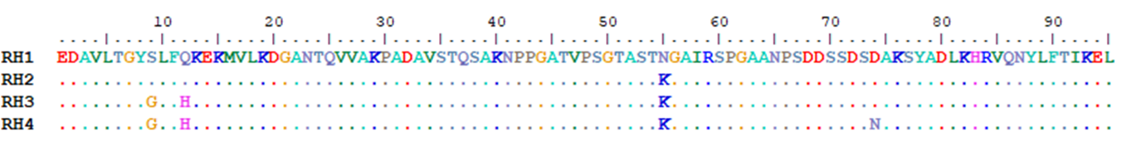
**

**Fig. S2 Sequence alignment of MSP1 haplotypes.**

E1 and E2 are the family-specific regions, and R1 is the tri-peptide repetitive region in K1 and MAD20 allelic families. The RO33 haplotypes are unique sequences without tandem repeats.


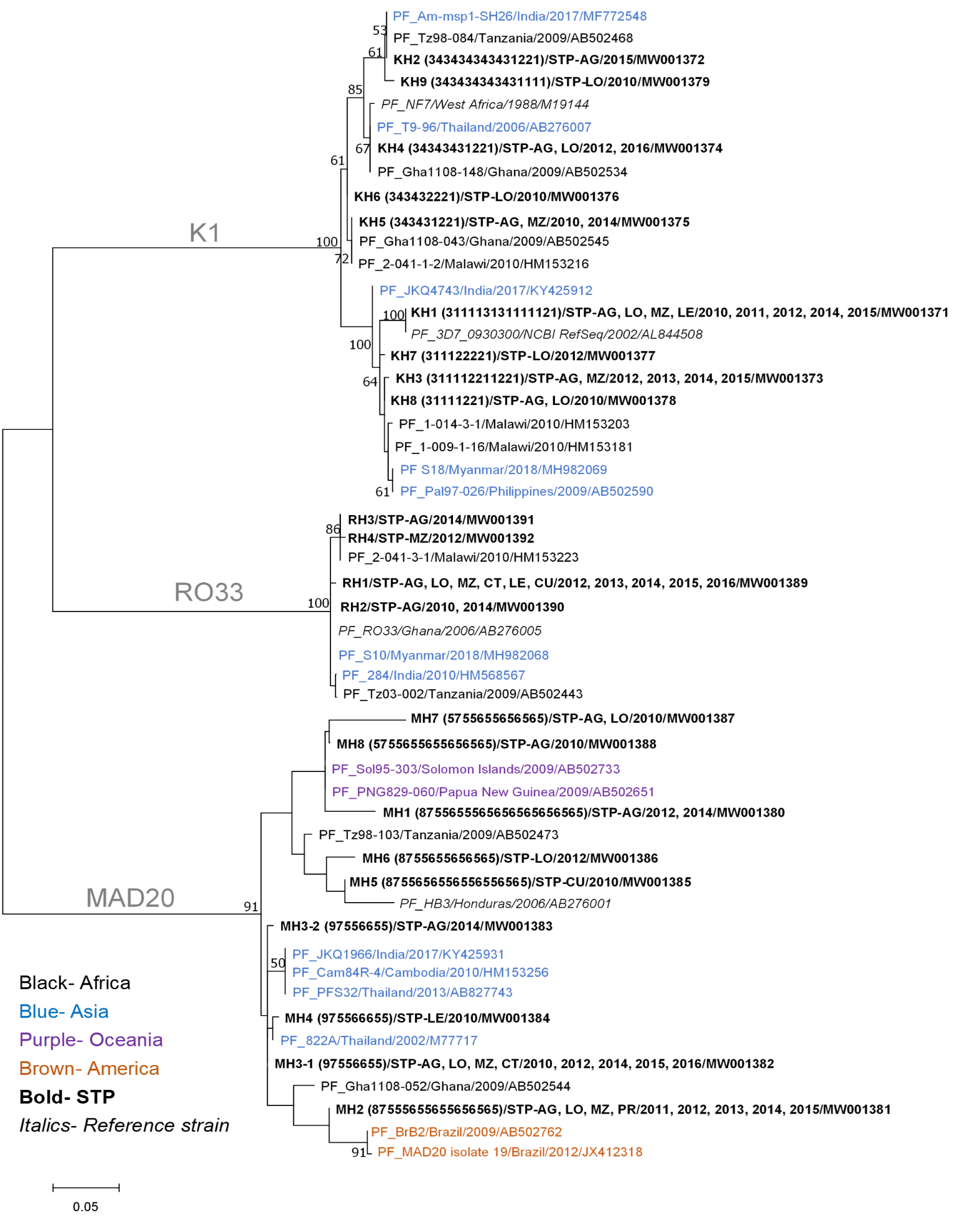


**Fig. S3 Phylogenetic tree of the *msp1* sequences from STP and other countries.**

Sequences are denoted by *P. falciparum* isolate/Origin (Country-District)/Year/Accession number. SGT, SGP, SAQ, and SGA repeats presented in the K1 haplotypes are denoted as 1, 2, 3, and 4 in the parentheses after the isolate. SGG, SVA, SVT, SKG, and SSG repeats presented in the MAD20 haplotypes are denoted as 5, 6, 7, 8, and 9, respectively. The bootstrap value below 50 is not shown on the tree. MH3-1 and 3-2 are the two nucleotide sequences that can be translated to the identical protein sequence MH3.

**(A) 3D7/IC family**

**
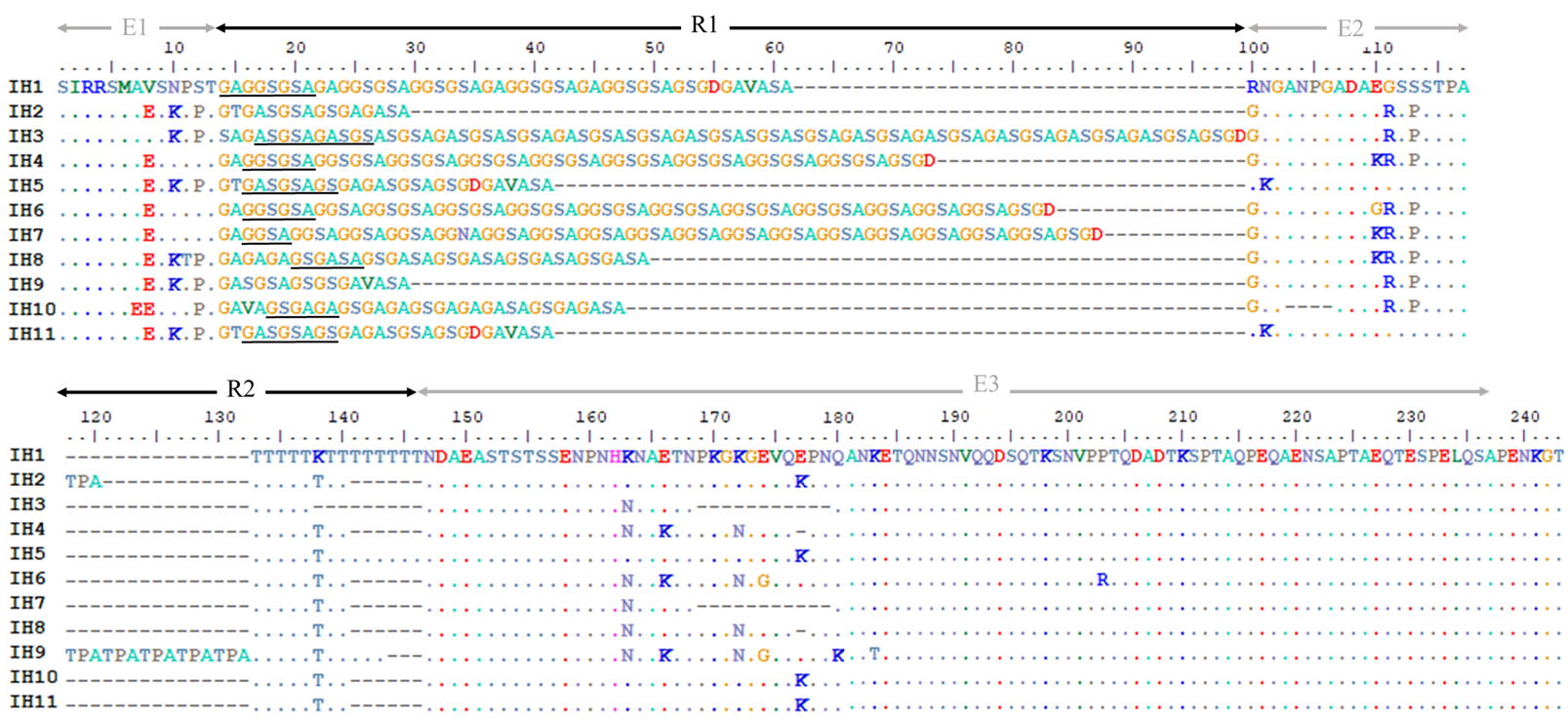
**

**(B) FC27 family**

**
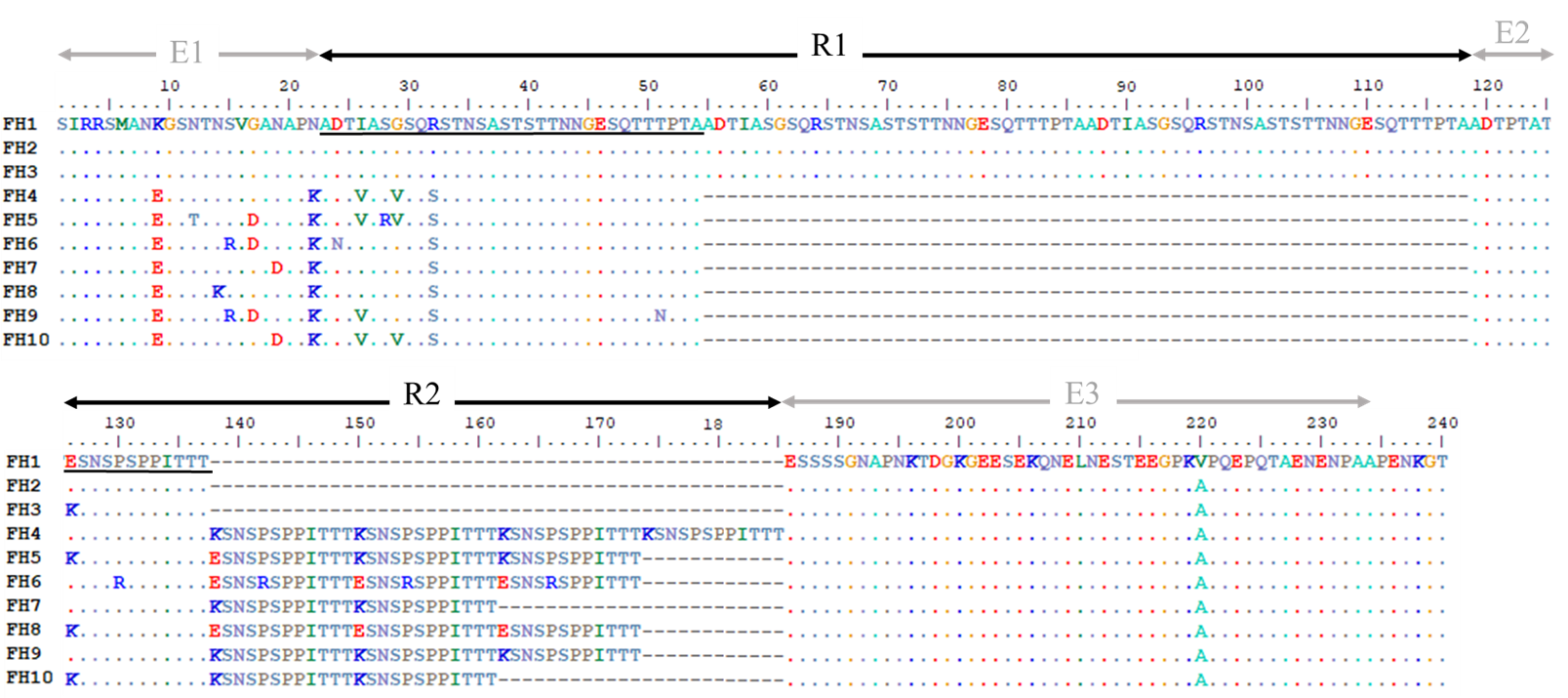
**

**Fig. S4 Sequence alignment of MSP2 haplotypes.**

The family-specific regions are E1-E3, and the two tandem repetitive regions are R1 and R2. Each repeat unit in R1 and R2 is underlined.

**
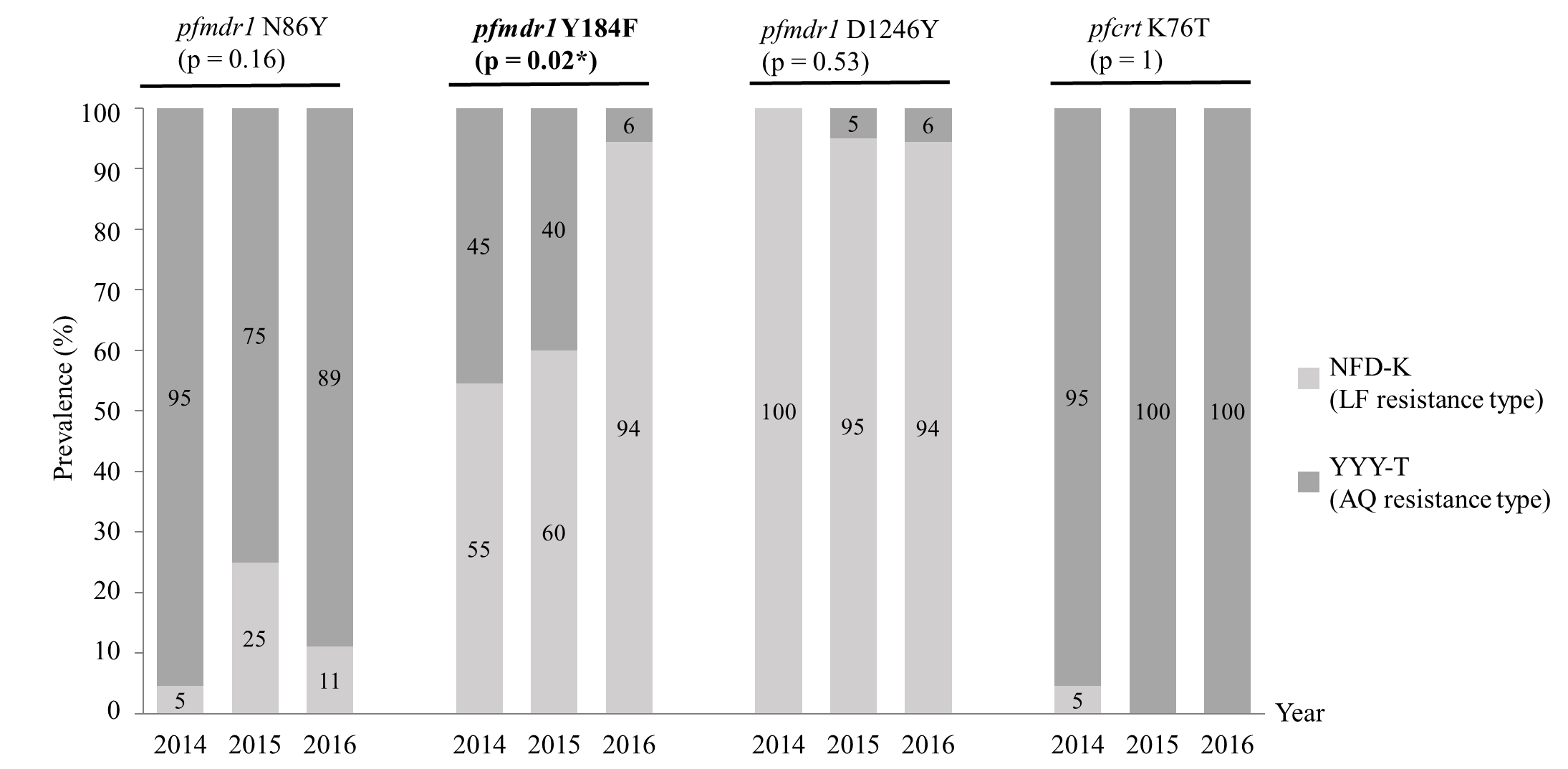
**

**Fig. S5 Temporal changes of *pfmdr1* and *pfcrt* polymorphisms in 60 pre-treatment samples from 2014 to 2016.**

The figures show temporal changes of *pfmdr1* and *pfcrt* polymorphisms in 60 pre-treatment samples, of which 22 samples are collected from 2014, 20 samples from 2015, and 18 samples from 2016. Significant p-values from Fisher’s exact tests are shown in bold type. Values in the bar graph are prevalence (%).

**(A)**

**
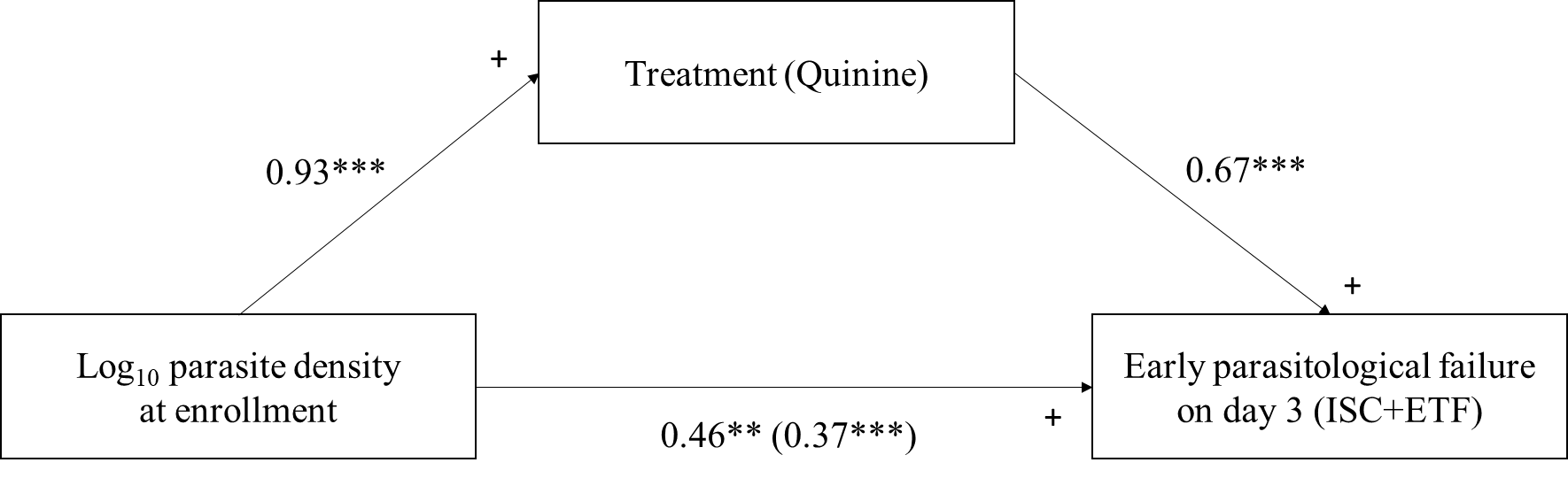
**

**(B)**

**
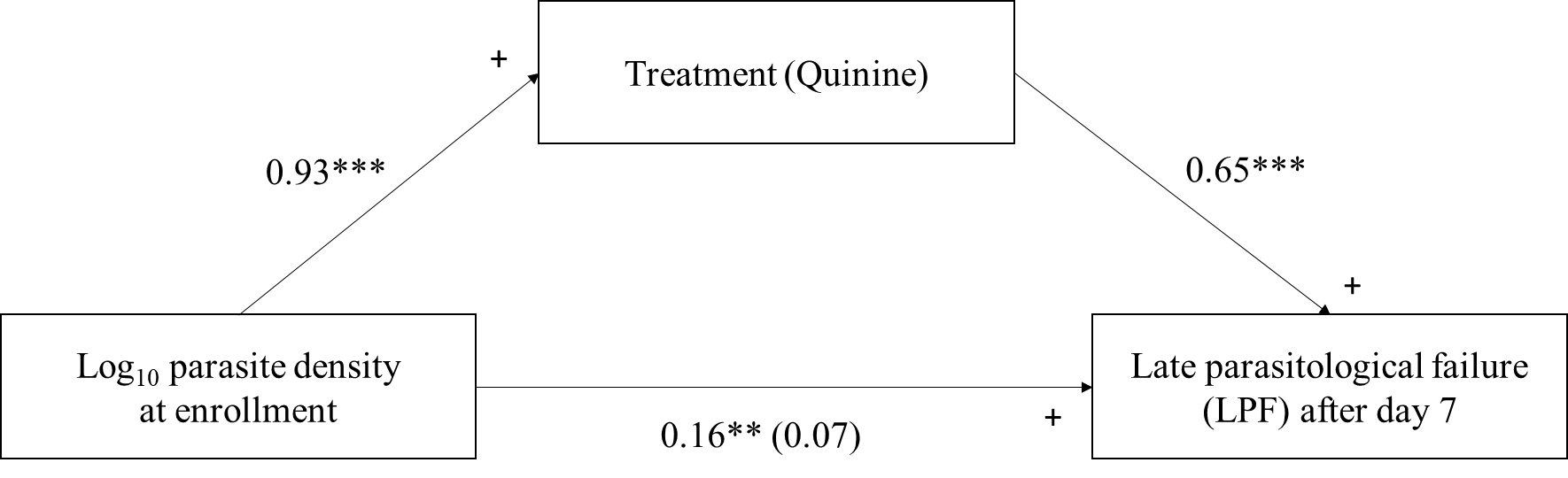
**

**Fig. S6 Relationship between initial parasite density, treatment types, and parasitological treatment failures**

(A) the mediation pathway of parasite density → treatment type (Quinine or ACT) → early parasitological failure on day 3 (including insufficient clearance and early treatment failure) after adjusting other predictive factors in the model. (B) the mediation pathway for the outcome of late parasitological failure after day 7. Number without parentheses is the coefficient of direct association, and number in the parentheses is the coefficient after adjusting treatment effect (+ positive association, **p < 0.01, ***p < 0.001). The initial parasite density is a significant factor to early parasitological failure, but no longer significant to the late parasiotological failure after adjusting treatment types.

**Table S1. Substitutions of *pfmdr1* and *pfcrt* genotypes after treatment in seven recurrent infections**

**
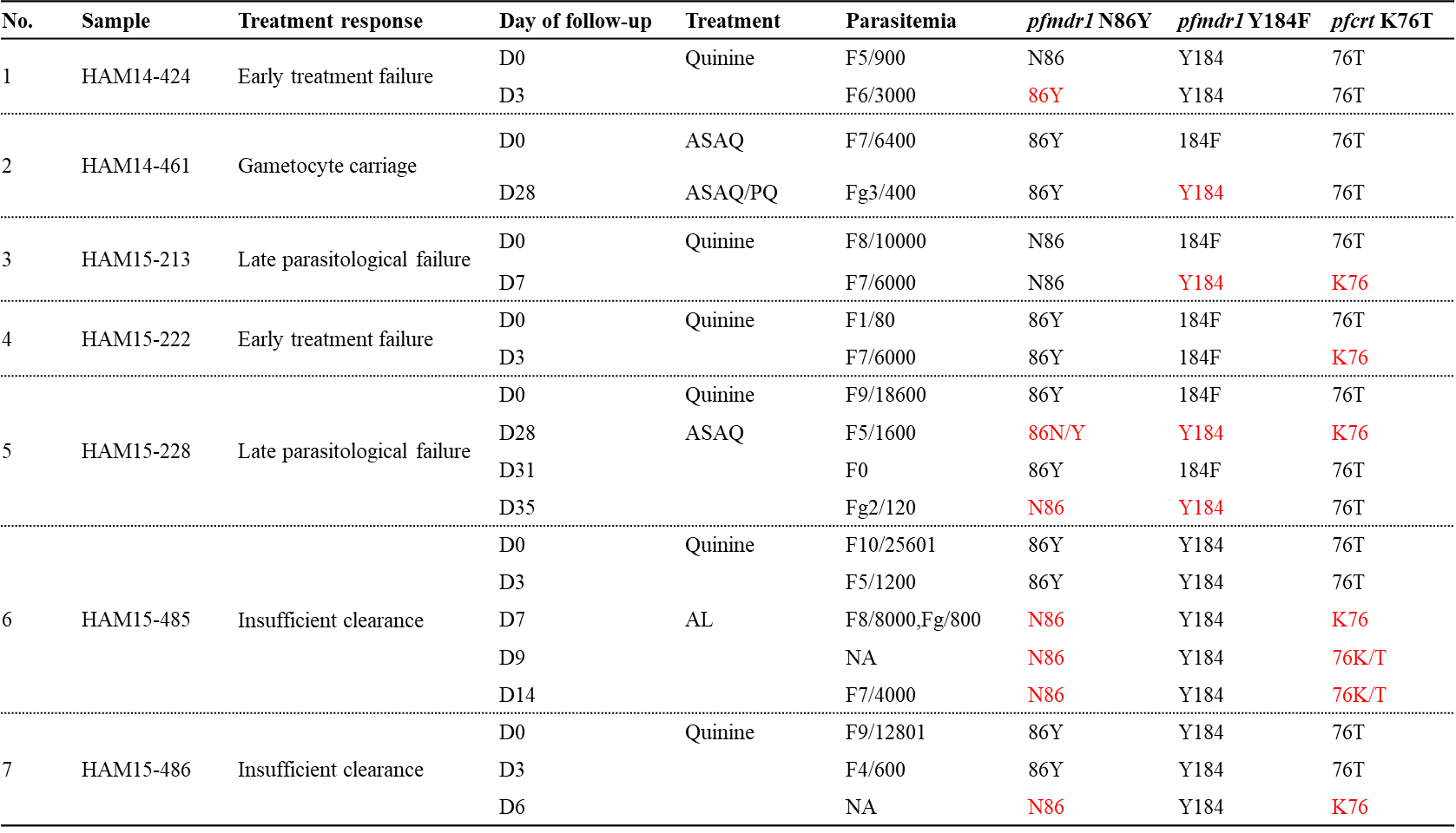
**

ASAQ = artesunate**-**amodiaquine, PQ = primaquine, AL = artemether-lumefantrine, NA = Not applicable. Substitutions of *pfmdr1* and *pfcrt* alleles after treatment are shown in red.
